# A comprehensive overview of triptolide utilizing nanotechnology and its potential applications in prostate diseases

**DOI:** 10.3389/fphar.2025.1592066

**Published:** 2025-06-17

**Authors:** Tongyin Yan, Yiao Wang, Zhiyan Hou, Pan Song, Puze Wang, Jinze Li, Jing Zheng, Dong Lv

**Affiliations:** ^1^ Department of Pharmacy, the First Affiliated Hospital of Zhengzhou University, Zhengzhou, China; ^2^ College of Pharmacy, Zhengzhou University, Zhengzhou, China; ^3^ Department of Urology, Institute of Urology, West China Hospital of Sichuan University, Chengdu, Sichuan, China; ^4^ Department of Urology, People’s Hospital of Deyang City, Chengdu University of Traditional Chinese Medicine, Chengdu, Sichuan, China; ^5^ Department of Anesthesia and Operating Room, Sichuan Provincial People’s Hospital, School of Medicine, University of Electronic Science and Technology of China, Chengdu, Sichuan, China

**Keywords:** triptolide, nanotechnology, prostate diseases, anti-inflammatory, anti-tumor

## Abstract

Triptolide (TPL) demonstrates a broad spectrum of biological and pharmacological activities, with its primary effects encompassing anti-inflammatory and anti-tumor properties, thereby rendering it applicable in the treatment of various diseases. However, the toxicity associated with TPL has considerably limited its clinical application. In recent years, the advancement of functional nanotechnology has created new opportunities for the application of TPL. TPL has been formulated using nanotechnology, resulting in a stable and tightly bound preparation. Regarding nanoparticle release, TPL can rapidly release the drug in acidic environments, such as tumor tissues, through pH-sensitive nanoparticles, while releasing the drug slowly under normal pH conditions. Furthermore, the surface characteristics and particle size of the carrier can be adjusted to control the drug release rate, thereby enhancing efficacy and reducing side effects. In terms of nanotargeting, active targeting achieved through surface modification can increase the concentration of the drug at the lesion site. Nanotechnology enhances the effectiveness of TPL, underscores its clinical advantages and potential, improves its disease-related performance, and offers novel strategies for disease treatment. This strategy is essential for improving therapeutic efficacy while minimizing side effects and enhancing bioavailability. Nano-TPL exhibits considerable potential for clinical application, owing to its effective targeted anti-inflammatory and anti-tumor properties, as well as its minimal toxic side effects. In this review, we present a succinct summary of the pharmacological activities and adverse effects of TPL, modifications made to its delivery system *via* nanotechnology, and its clinical application prospect is exemplified by prostate disease.

## Introduction

*Tripterygium wilfordii Hook F* (TwHF) is a species belonging to the Celastraceae family, characterized by perennial, vine-like growth patterns. This plant is indigenous to the southern regions of China, specifically along the Yongzhi River, as well as in Fujian Province and Taiwan. Renowned for its extensive therapeutic applications, TwHF is a traditional Chinese medicinal herb primarily utilized in the management of autoimmune and inflammatory conditions. Its pharmacological efficacy is attributed to a variety of bioactive constituents, including sesquiterpene pyridine alkaloids and celastrol, which demonstrate pronounced anti-inflammatory and immunomodulatory properties. Historically, TwHF has been employed both as a botanical insecticide and in traditional medicinal practices for ailments such as rheumatoid arthritis and systemic lupus erythematosus (Shan et al., 2023; Zhang X. et al., 2024). Tripterygium has numerous therapeutic applications, primarily in the treatment of RA, cancer, and IgA nephropathy. In the context of RA treatment, the plant and its components—such as TPL (Triptolide), celastrol, and tripterygium glycosides—exhibit anti-inflammatory effects and can inhibit bone destruction, thereby alleviating RA symptoms and enhancing patient quality of life (Sun et al., 2020). Regarding cancer treatment, TPL, a key bioactive component, has demonstrated significant activity against various cancers, capable of inhibiting growth in all 60 U.S. National Cancer Institute cell lines at low doses and inducing cell death (Lim et al., 2021). For IgA nephropathy, a polyglycoside preparation derived from the plant has proven effective by reducing proteinuria, lowering blood creatinine levels, and protecting kidney function. Additionally, Tripterygium vine offers several advantages in the treatment process. Its compounds target multiple pathways, providing a broad therapeutic scope that is beneficial for complex diseases such as RA and cancer (Bai et al., 2020). Furthermore, as a natural product, it presents an alternative to synthetic drugs, potentially resulting in fewer side effects and improved tolerability. Moreover, active compounds like TPL show promise in the development of new anticancer drugs due to their ability to interfere with cellular gene regulation. However, the application of TwHF in traditional medicine necessitates careful consideration of its potential adverse effects, notably concerning its toxicity profiles, which include hepatotoxicity and nephrotoxicity.

With the ability to provide exceptional sensitivity, precision, efficiency, adaptability, and high throughput, nanotechnology has profoundly influenced the field of medicine. As nano-oncology emerges as a key discipline in oncology research and clinical practices, there is an urgent necessity for the advancement of novel nanotechnology-based analytical tools, diagnostic techniques, and therapeutic approaches. These innovations can offer valuable insights into carcinogenesis at a molecular level while facilitating personalized treatment strategies tailored to the unique genetic and proteomic characteristics of individual patients (Tenchov et al., 2025). The application of TwHF nanotechnology showcases an innovative method to amplify the therapeutic effects of this traditional Chinese medicinal plant. Known for its anti-inflammatory and immunomodulatory properties, TwHF’s clinical utility is often hindered by its toxicity. The integration of TwHF with nanotechnology presents multiple benefits, particularly in enhancing therapeutic efficacy and safety. The goal of nanotechnology applications is to optimize the efficacy of TwHF while concurrently minimizing adverse reactions. This synergy can lead to the development of advanced drug delivery systems that lower side effects, thereby increasing the effectiveness of TwHF in treating diverse medical conditions. The respect of enhancing drug delivery and therapeutic impact *via* the integration of TwHF with nanotechnology is significant. Nanoparticles can enhance the bioavailability of active compounds in TwHF, such as celastrol, by allowing targeted delivery to affected tissues (Pei et al., 2021). This meticulous delivery approach can mitigate systemic toxicity, yielding safer and more effective treatment outcomes (Sun et al., 2022). To elevate the safety profile and reduce the toxicity associated with TwHF, it is essential to combine it with nanotechnology. The use of nanocarriers can significantly decrease the toxicity related to the oral administration of TwHF extracts, as indicated by formulations, like eye drops, that maintain safety and stability (Zhu et al., 2025). Nanotechnology supports the controlled release of active compounds, helping to lower adverse effects while sustaining therapeutic levels over time (Gressler et al., 2025). Currently, TwHF nanotechnology has displayed potential in treating conditions such as RA and kidney diseases, with ongoing research concentrating on its mechanisms and safety profiles (Zhang et al., 2021; Tong et al., 2022). By merging nanotechnology within TwHF formulations, new therapeutic strategies can be developed that capitalize on their intricate metabolite characteristics while addressing essential safety concerns (Ren et al., 2021). The combination of TwHF and nanotechnology is widely employed in the treatment of various diseases, including RA, systemic lupus erythematosus, cancer, diabetic nephropathy, liver fibrosis, psoriasis, allergic reactions, and various malignant tumors (Zhao et al., 2021). Prostate diseases represent a continuous concern that nearly every man encounters throughout his life, encompassing conditions such as prostatitis in younger years and progressing to prostatic hyperplasia and prostate cancer in later stages of life. Inflammation, immunity including autoimmune, and tumor cell progression play important roles in the pathogenesis and progression of prostate diseases. Given its potent anti-inflammatory, anti-tumor and immunomodulatory properties, TwHF has demonstrated considerable application potential in the treatment of prostate diseases. This study provides a comprehensive review of the characteristics and mechanisms of TwHF, analyzes the current research status of nano-TwHF, and evaluates its potential application value in prostate diseases.

## Pharmacological effects of TPL

### Pharmacological activity

In recent years, numerous researchers have examined the primary active constituents of TwHF, specifically celastrol, TPL, and triptonide, which are three sesquiterpene lactones predominantly sourced from the roots of TwHF(Wong et al., 2012). Each of these compounds possesses distinct chemical structures, with variations primarily evident in their substituent groups and their respective positions on the cyclic framework (Hou et al., 2019). Compared with TPL, triptonide is structurally very similar, differing only in the C-14 substituent (Song J. et al., 2023). However, there are fundamental distinctions among these compounds. They demonstrate significant pharmacological activities; foremost among these is their anti-inflammatory capability, predominantly attributed to TPL (Ji et al., 2025) and celastrol (Tan et al., 2024). This activity occurs through the inhibition of the nuclear transcription factor NF-κB and NLRP3 (Li D. et al., 2025), leading to reduced production of pro-inflammatory cytokines such as tumor necrosis factor-α (TNF-α) and interleukin-1β (IL-1β). In the clinical trial research carried out by Ren et al. (2007), it was evidently demonstrated that in the process of utilizing TwHF for the treatment of inflammatory bowel diseases, the levels of serum C-reactive protein (CRP), TNF-α, and IL-1β were remarkably reduced. This reduction was achieved through the inhibition of the nuclear transcription factor NF-κB, which plays a crucial role in the inflammatory pathway. The results of this study contribute significantly to understanding the potential therapeutic mechanism of TwHF in managing inflammatory bowel diseases and offer valuable insights for further exploration and development of more effective treatment strategies. Furthermore, in *Mycobacterium tuberculosis* (Mtb)-infected macrophages, tretinoin upregulated the expression of lincRNA-p21, while down-regulating the expression of lncRNA-PACER. The regulation of these lncRNAs was associated with changes in the expression of pro-inflammatory genes Ptgs-2 and IL-6 (Tamgue et al., 2021). It has the potential to impact various inflammation-related signaling cascades, including JAK/STAT (Huang et al., 2023), MAPK/ERK (Yanchun et al., 2019), AMPK(Huang et al., 2021) and PI3K/Akt (Yang et al., 2022), effectively suppressing the inflammatory process from multiple avenues. The second notable effect is immunosuppression. TwHF not only curtails hyperactive immune responses but also enhances immunity to an extent, particularly by inhibiting the activities of immune cells such as T lymphocytes (Xie et al., 2024; Wu et al., 2025), B cells, and macrophages, along with the release of inflammatory mediators such as TNF-α and IL-1β. Both TPL and celastrol can suppress the synthesis of pro-inflammatory cytokines, stimulate the production of anti-inflammatory cytokines, and expedite the apoptosis of certain immune cells, including activated T cells and B cells. They facilitate the differentiation of immune cells, with TwHF predominantly inhibiting the differentiation of Th1 and Th17 cells (Fu et al., 2021), while inhibiting M1-type alveolar macrophage (AM) polarization and promotion of M2-type macrophage polarization reduces inflammatory cell infiltration and cupular cell proliferation in the airway (Liang et al., 2024). Lastly, the anti-tumor properties are noteworthy. Celastrol and TPL are the main active compounds from TwHF that have anticancer activity (Alam et al., 2021). TPL has been shown to activate both intrinsic and extrinsic apoptotic pathways, triggering the Caspase cascade (Xu T. et al., 2023), leading to apoptosis. Based on the clinical research conducted by Kitzen, J. J. E. M., and other investigators (Kitzen et al., 2009), it has been demonstrated that during the treatment of advanced cancer conditions, TPL has the capacity to induce an elevation in Caspase-3 activity. Concurrently, this compound also prompts a significant apoptosis phenomenon among monocytes and neutrophils. Such findings provide valuable insights into the potential mechanisms and effects of TPL in the context of advanced cancer therapeutics. It directly influences tumor cell cycles, thereby impeding tumor cell proliferation (Wang et al., 2025). Tumor tissue often depends on neovascularization for nutrient and oxygen acquisition. The constituents of TwHF can diminish the expression of vascular endothelial growth factor (VEGF) (Li Z. et al., 2022), interfere with vascular endothelial cell migration and proliferation, thus severing the blood supply to tumors and impeding angiogenesis (Song M. et al., 2023). Additionally, certain studies indicate TwHF possesses antifungal, antibacterial (Lu et al., 2023), and antiviral (Aliabadi et al., 2022; Chen et al., 2023) properties. Celastrol, the active ingredient inTwHF, possesses potent antibacterial effects, especially against methicillin-resistant *Staphylococcus aureus* (MRSA). It exhibits excellent anti-MRSA activity by targeting Δ1-pyrroline-5-carboxylate dehydrogenase (P5CDH) to inhibit bacterial growth, induce oxidative stress and inhibit DNA synthesis (Yuan et al., 2023). Other components of TwHF have also been found to have anti-bacterial activity (Abdel-Halim et al., 2022). It demonstrates inhibitory effects on a range of microorganisms and may be employed to combat infections stemming from bacteria or viruses. Evidence has suggested that celastrol and celastrol derivatives inhibits various viruses, including the hepatitis B virus (Zhang and Lu, 2020).

Numerous clinical trials investigating TwHF have been conducted, focusing on a variety of conditions such as RA, systemic lupus erythematosus, IgA nephropathy, renal transplant rejection, inflammatory bowel disease, gastrointestinal cancer, gastric cancer, ect. Lv et al. (2015) conducted a phase III clinical trial involving 207 patients with RA to evaluate the efficacy of TwHF in comparison to methotrexate (MTX). The findings demonstrated that the therapeutic effectiveness of TwHF for treating RA was comparable to that of MTX. Wang et al. conducted a Phase I clinical trial involving 80 renal transplant patients. The incidence of transplant rejection was significantly lower in the TwHF group (15.0%) compared to that in the conventional treatment group. In another phase I clinical trial (Ren et al., 2007) with 65 inflammatory bowel disease patients. Significant reductions in serum CRP, TNF-α, and IL-1β levels were found in the patients undergoing treatment with TwHF. A phase I clinical study conducted by Kitzen et al. (2009) demonstrated that the novel cancer cell apoptosis inducer F60008 can be converted into TPL, thereby activating tumor cell apoptosis *in vivo* in patients with advanced solid tumors. However, this process is associated with increased caspase-3 activity and the apoptosis of monocytes and neutrophils. The clinical trials of TwHF were summarized in Table 1.

**TABLE 1:** The clinical trials involving TwHF.

Author	Clinical trials	Disease	Participants (n)	Comparator	Primary endpoint	Outcomes	Adverse events
Lv et al. (2015)	Phase Ⅲ: completed	RA	207	MTX	Radiologic data and clinical remission rates	TwHF had comparable efficacy with MTX in the treatment of RA.	Gastrointestinal symptoms, reversible amenorrhea (perimenopausal women).
Liu, et al. (2014)	Phase Ⅰ: completed	Systemic lupus erythematosus (SLE)	79	Prednisone acetate + MTX	Changes after 6 months of treatment:24-h urine protein, Serum C3 level, SLEDAI score	The effective rate of TwHF combined with prednisone in the treatment of SLE was 87.5%, surpassing that of prednisone combined with MTX.	Higher incidence of menstrual disorders in the observation group; no significant differences in nausea/vomiting or upper respiratory infections.
Liang et al. (2019)	Phase Ⅲ: completed	IgA nephropathy	128	Tripterygium glycosides, Irbesartan	Changes after 12 weeks of treatment: 24-h urinary protein, serum albumin (Alb), estimated glomerular filtration rate (eGFR), urinary podocyte count	This combination therapy has a synergistic protective effect against renal podocyte injury and disease progression.	Mild gastrointestinal disturbances; no severe adverse events reported.
Chen et al. (2015)	Phase Ⅲ: completed	IgA nephropathy	20	Mycophenolate Mofetil (MMF) monotherapy	Clinical efficacy; renal function markers: 24-h urinary protein, blood urea nitrogen (BUN), serum albumin (Alb), serum creatinine (SCr)	The recurrence rate was significantly reduced.	Infections, diarrhea, vomiting, leukopenia, and liver dysfunction; no severe adverse events.
Wang et al. (2015)	Phase Ⅰ: completed	Rejection after kidney transplantation	80	Cyclosporine (CsA) monotherapy	Incidence of rejection; renal function markers (serum creatinine); drug safety	There were no instances of acute rejection in the experimental group, and the rejection rate within 6 months was 2.5%, markedly lower than the 15.0% observed in the control group.	Lower infection rate; fewer adverse events in TG group.
Ren et al. (2007)	Phase Ⅰ: completed	Inflammatory bowel diseases	65	No control (single-arm trial)	Clinical remission rate; endoscopic improvement; change in inflammatory markers	Serum CRP, TNF-α and IL-1β levels were significantly decreased.	Mild adverse reactions: diarrhea, headache, nausea, facial rash (self-resolving).
Borazanci et al. (2024)	Phase Ⅰ: ongoing	Gastrointestinal Cancer	45	None (single-arm study)	Safety (MTD, DLTs); pharmacokinetics (PK) of Minnelide/TPL; antitumor activity (RECIST 1.1, Choi criteria)	The primary toxicity experienced was hematologic.	Grade ≥3 toxicity Most common: neutropenia, severe cerebellar toxicity; no treatment-related deaths.
Lim et al. (2024)	Phase Ⅰ: ongoing	Gastric cancer	36	Monotherapy vs combination	MTD, DLT, and antitumor activity assessment	Minnelide might overcome resistance to paclitaxel and enhance the sensitivity.	Common: neutropenia, abdominal pain, nausea; grade ≥3: neutropenia, abdominal pain, nausea.
Wu et al. (2013)	Phase Ⅰ: completed	Henoch-Schönlein purpura	56	Prednisone alone	Short-term remission and long-term prognosis	TPL is effective in relieving short-term symptoms for moderately severe HSPN children.	Liver injury, leukocytopenia, hypertension, hyperglycemia.
Wu and Guo (2005)	Phase Ⅰ: completed	Psoriasis vulgaris	103	No control (single-arm trial)	PASI improvement	Only in few patients with decreased WBC during the treatment period.	Leukocytopenia, proteinuria, menstrual cycle delay.
Kitzen et al. (2009)	Phase Ⅰ: completed	Advanced Cancer	20	No control (single-arm trial)	MTD, DLT, pharmacokinetics, pharmacodynamics	Caspase-3 activity increased, and monocytes and neutrophils obviously apoptosis.	Grade 4 neutropenia, lethal events, mild anemia, fatigue, nausea.
Chen et al. (2014)	Phase Ⅲ: completed	Autosomal Dominant Polycystic Kidney Disease	100	No control (single-arm trial)	Antiproteinuric effect, TKV inhibition, eGFR stabilization	Inhibit the cyst formation and growth in ADPKD models.	Menstrual abnormalities, amenorrhea, no bone marrow suppression or liver toxicity.

### Toxicity

The toxicity associated with TwHF is a significant concern (Song J. et al., 2023). Celastrol and TPL have potential hepatotoxicity (Hu et al., 2022; Liu et al., 2025). Numerous studies have focused on its hepatotoxicity, which primarily presents as abnormal liver function, triptotriterpenic acid A and triptobenzene H might be the main hepatotoxic components of TwHF(Li et al., 2023). The underlying mechanisms are linked to oxidative stress, DNA damage, mitochondrial dysfunction, endoplasmic reticulum stress (ERS), and various contributing factors (Fu et al., 2013). The hepatotoxicity caused by celastrol is directly related to its accumulation in the liver cells (Jin et al., 2019). A central mechanism involves a substantial increase in intracellular reactive oxygen species (ROS) levels (Jin et al., 2019), triggered by a vigorous oxidative stress response. These ROS’s high chemical reactivity allows them to target lipids, proteins, and DNA within cell membranes, resulting in severe consequences such as lipid peroxidation of cell membranes, loss of protein functionality, and DNA strand breaks (Zhang et al., 2018; Liu et al., 2020). TPL also jeopardizes the integrity of both inner and outer mitochondrial membranes, leading to diminished mitochondrial membrane potential, lower ATP synthesis efficiency, and inadequate cellular energy supply (Zhang et al., 2022). Notably, this mitochondrial compromise further escalates ROS production, creating a positive feedback loop that intensifies cellular damage. Once the damage surpasses a specific threshold, the equilibrium between apoptosis and autophagy is disrupted, culminating in structural and functional degradation of liver tissue (Fu et al., 2011; Hasnat et al., 2019). The ensuing hepatocyte injury and death release various inflammatory mediators, activate hepatic innate immune cells such as Kupffer cells (Su, 2002), and instigate localized inflammatory responses. The excessive buildup of inflammatory mediators not only exacerbates liver cell damage but may also propagate to surrounding healthy cells *via* the bystander effect, widening the damage scope (Abrignani, 1997; Kim et al., 2018). In addition to hepatotoxicity, TwHF presents nephrotoxic risks (Zhang et al., 2016), which may manifest as acute or chronic kidney impairment, potentially resulting in renal failure in severe cases (Feng et al., 2019). Particularly, TPL has been shown to directly harm renal tubular epithelial cells, inducing tubulointerstitial nephritis (Jiang et al., 2023). The impairment of renal tubular epithelial cells is frequently accompanied by alterations in cell membrane permeability, leading to protein leakage and further detriment to overall renal function (Li et al., 2014; Tong et al., 2022; Jiang et al., 2023).

The aforementioned toxicities of TwHF are frequently associated with dosage and duration of use. What we must acknowledge is the “dual nature” of TPL, which encompasses both its remarkable therapeutic benefits and its potential toxicities. On one hand, the adverse effects and toxicity associated with TPL can impact multiple physiological systems, particularly the hepatic system, with a narrow therapeutic index. Prolonged or excessive administration can exacerbate toxic reactions due to the accumulation of the compound within the body. Conversely, TPL’s anti-inflammatory and anti-cancer properties are capable of effectively targeting hepatic cancer cells, inhibiting their proliferation, and providing protective effects on liver health. This presents both a challenge and an opportunity, necessitating a dialectical approach in assessing whether TPL serves as an adversary or an ally.

Although TPL exhibits significant antitumor and anti-inflammatory activities, its clinical application is limited by multi-organ toxicity—mainly hepatotoxicity and nephrotoxicity—a narrow therapeutic window, and poor physicochemical properties such as low water solubility and a short half-life. Studies have shown that the toxicological mechanisms of TPL involve mitochondrial dysfunction (Xing et al., 2024), oxidative stress (Li C. et al., 2022; Cao et al., 2024), and dose-dependent effects, where low doses (125 μg/kg) provide neuroprotective benefits, while high doses (500 μg/kg) worsen tissue injury (Sanadgol et al., 2018). To overcome these limitations, researchers have explored several multidimensional strategies. Nanotechnology-based approaches, including pH-responsive nanoparticles (Chang et al., 2024; Yu et al., 2024), biomimetic nanocarriers (Li Z. et al., 2024; Yu et al., 2024), and antibody-drug conjugates (Wang et al., 2024), have been developed to improve the balance between efficacy and toxicity by increasing drug accumulation at tumor sites up to 2.7-fold and reducing systemic exposure. In terms of dose optimization, nanoparticle formulations have achieved effective RA treatment at doses as low as 0.075 mg/kg (Liu et al., 2021), representing a greater than 90% reduction compared with traditional formulations. In addition, combination therapies such as co-administration with glycyrrhizic acid (Cao et al., 2024) have significantly reduced hepatotoxicity by activating the Nrf2 pathway, lowering ALT and AST levels by 40%–60%. However, most of these strategies remain in the preclinical stage, and several challenges still need to be addressed, including the large-scale manufacturing stability of targeted delivery systems, potential metabolic interactions in combination therapies, and the need for individualized dose adjustment. Overall, advances in precise drug delivery and intelligent release systems, combined with dynamic dose regulation and synergistic therapies, provide a promising direction for the clinical translation of TPL.

### Nanotechnology-based TPL

The clinical application of TPL is limited by several factors including its significant systemic toxicity and poor water solubility. Recent researches have been intensely focused on identifying strategies to overcome these drawbacks of TPL. The advent of nanotechnology offers innovative strategies for enhancing the utilization of TPL. The physicochemical properties of nanoparticles, such as shape, size, and surface chemistry, play a crucial role in their ability to penetrate tumors. Optimizing these characteristics can improve their transport, leading to enhanced accumulation and retention within tumor sites (Wen et al., 2022). The nano-formulation of TPL (TP-LCNPS-GEL) utilizes a lipid cubic crystalline nanoparticle gel-based transdermal delivery system to significantly enhance drug permeability and bioavailability. By leveraging its sustained-release properties, it prolongs therapeutic duration while amplifying dual anti-inflammatory and bone-repair effects, and reduces systemic toxicity through targeted controlled-release mechanisms, offering a high-efficacy, low-toxicity strategy for RA therapy (Pei et al., 2022). The application of TPL in nanotechnology can be categorized into three primary aspects: nano-drug carriers, nano-controlled release systems, and targeting strategies. By enhancing water solubility, improving bioavailability, enabling precise targeting, optimizing controlled release systems, and minimizing toxic side effects, it paves the way for the advancement and application of TPL.

## Nano-drug carriers

**FIGURE 1:**
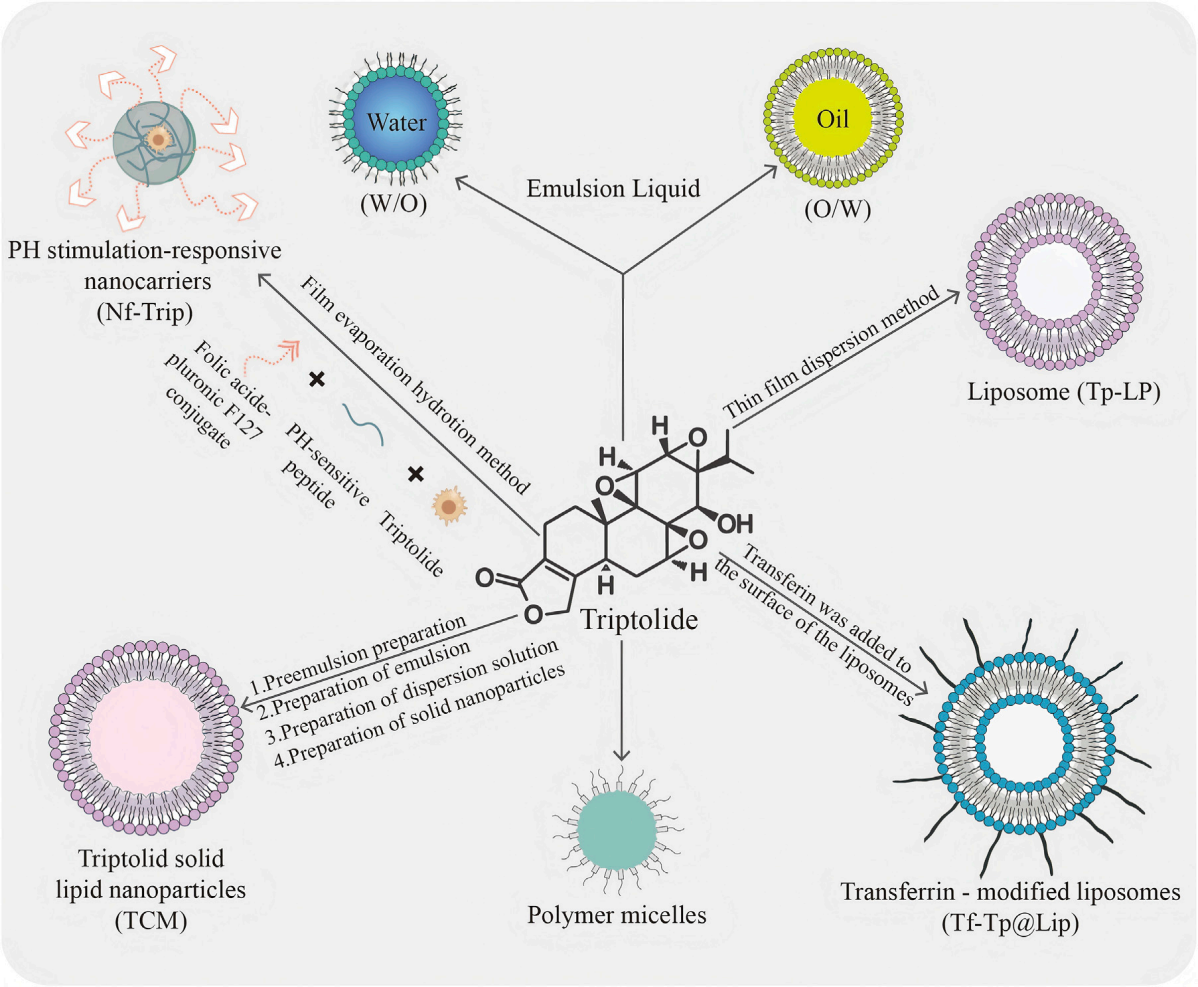
Schematic representation of different types of commonly used TPL nanocarriers. The diagram illustrates various types of commonly used TPL nanocarriers: TPL preparation of hydrophilic and lipophilic emulsions; Formation of liposomes (TP-LP) *via* the thin film dispersion method; Modification of liposomes with transferrin on their surface to create transferrin-modified liposomes (Tf-Tp@Lip); Development of polymeric micelles; Synthesis of TPL solid lipid nanoparticles (TCM) through a process involving pre-emulsion, emulsion, dispersion, and solid nanoparticles; and Creation of pH-stimulated responsive nanocarriers using a specific polymer through film evaporation hydration.

Nanotechnology has been actively utilized in the development of drug delivery systems that overcome the limitations of conventional carriers (Al Ayidh et al., 2025). Nanocarriers significantly enhance the stability and bioavailability of drugs by encapsulating TPL within or adsorbing it onto the surface of nanoparticles. The most recent nanocarriers can be classified into liposomes, polymeric nanoparticles, and solid lipid nanoparticles. By encapsulating TPL, nanocarriers can protect the compounds from degradation in the biological environment (Lin et al., 2023). Furthermore, they can enhance the solubility and stability of TPL, leading to improved absorption and bioavailability (Qiu et al., 2023). The effects of nanotechnology on TPL can be categorized into several key aspects (Figure 1). The first objective is to improve water solubility and stability. A core advantage of nanotechnology lies in its ability to enhance the water solubility and stability of drugs. The use of nano-drug delivery systems modifies the particle size of natural products, thereby increasing drug solubility and enhancing cellular or tissue absorption (Lv et al., 2024). TPL has been encapsulated or adsorbed onto the surfaces of nanoparticles, such as liposomes and polymer nanoparticles, which significantly improves its solubility and dispersibility in water. This approach not only addresses the issue of TPL’s poor water solubility but also enhances its stability during storage and application, thereby enabling the development of efficient and stable nano-Chinese medicine formulations. The second objective is to enhance bioavailability; nanomodification has significantly increased the bioavailability of TPL. The small size and surface modifications of nanoparticles facilitate drug absorption in the gastrointestinal tract while minimizing drug degradation and excretion within the body. Furthermore, nanocrystals can protect the drug from degradation in the internal environment and extend its circulation time in the body, thus improving both bioavailability and therapeutic efficacy.

As one of the most advanced nanodelivery systems, liposomes represent a distinct category of nanoformulation that has been applied in clinical therapies, with particle sizes ranging from 50 to 1,000 nm (Torchilin, 2008). Their unique lipid bilayer architecture allows for the effective encapsulation of hydrophilic drugs within the core, while lipophilic compounds can be securely embedded between the lipid layers (El-Samaligy et al., 2006; Zhang et al., 2013). The characteristics of liposomes, such as amphiphilicity, biocompatibility, and biodegradability, render them exceptionally advantageous for the delivery of Traditional Chinese Medicine (TCM). Moreover, these carriers enhance therapeutic efficacy and safety, improve bioavailability, facilitate sustained release, and enable localized drug administration (Chen et al., 2025). Zhong et al. (2021) introduced an innovative biomimetic liposome, coated with erythrocyte membranes and co-loaded with TPL and celastrol (C + T/RBCm@Lip), which exhibits a remarkable ability to evade recognition and clearance by macrophages (Hu et al., 2023). Liposomes serve as competent and superior carriers capable of encapsulating TPL, thereby safeguarding it during administration *via* various routes and preventing damage to healthy tissues while treating a range of diseases. By acting as a carrier for TPL, liposomes further enhance its pharmacokinetics compared to free TPL, extending the drug’s *in vivo* duration, significantly reducing toxicity, and markedly improving therapeutic effects through a controlled release mechanism (Ren et al., 2021). Polymeric micelles, with a mean size ranging from 20 nm to 200 nm, consist of a hydrophobic inner core that provides a space for the encapsulation of poorly water-soluble drugs, and a hydrophilic outer shell or corona that protects the drugs from the aqueous environment (Gaucher et al., 2005; Hwang et al., 2020). As a classical drug carrier, polymeric micelles possess numerous advantages, including favorable physicochemical properties for tumor targeting *via* the enhanced permeability and retention (EPR) effect, as well as an appropriate size that minimizes renal excretion and filtration by interendothelial cells (Danhier et al., 2015; Cabral et al., 2018). TPL entrapped in nanoparticles exhibits *in vitro* release characterized by a typical biphasic phenomenon, consisting of an initial burst release followed by sustained release. Polymeric micelles, serving as practical carriers, particularly enhance TPL absorption by increasing solubility and bioavailability, protecting the encapsulated TPL from harsh environmental conditions, and facilitating controlled release at target sites. Additionally, they prolong residence time and inhibit efflux pumps to improve TPL accumulation. Compared to single-functional micelles, multifunctional TP-polymeric micelles demonstrate higher safety factors and more potent antitumor effects in both *in vitro* and *in vivo* studies (Ren et al., 2021). Solid lipid nanoparticles (SLNs) are nanocarriers composed of solid natural or synthetic lipids, typically exhibiting particle sizes ranging from 50 to 1,000 nm. In recent years, SLNs have garnered extensive attention due to their advantageous properties, including high drug loading capacity, broad applicability, controlled drug release, and favorable biosafety and stability (Mehnert and Mäder, 2001; Mei et al., 2005; Luo et al., 2021). Research indicates that TPL can exert a therapeutic effect by significantly increasing the levels of ROS. However, it is important to note that ROS can damage DNA and induce lipid peroxidation within cells, rendering TPL highly toxic to normal metabolic organs such as the liver and kidneys. Mei et al. prepared and characterized TPL-loaded SLNs, demonstrating through a series of experiments that TPL-SLNs effectively mitigate liver toxicity while maintaining anti-inflammatory activity (Mei et al., 2005). The encapsulation of TPL within SLNs resulted in a notable reduction in toxicity and enhancement of efficacy.

### Nano-controlled release systems

An additional advantage of nanoformulations is their capacity to facilitate the controlled release of co-delivered drugs. This precise release mechanism ensures timely and targeted delivery of the drug, thereby enhancing its concentration at the specific site of the lesion (Xiao et al., 2021). Consequently, this strategy optimizes the drug’s efficacy while minimizing potential harm to healthy cells (Liu et al., 2024). Furthermore, the controlled release also contributes to prolonging drug metabolism and excretion, resulting in a decreased frequency of administrations and improved patient compliance with the prescribed treatment regimen (Asadi et al., 2025). The application of controlled release systems holds significant promise for enhancing the regulation of drug release over time. These systems facilitate the traversal of drugs across physiological barriers and direct them to the intended site of action while minimizing exposure to other areas of the body (Mendes et al., 2023), thereby improving therapeutic efficacy and reducing the frequency of drug administration. Recent advancements in controlled release techniques include stimulus-responsive controlled release and continuous stable release. Stimulus-responsive controlled release utilizes stimuli-responsive polymers that enable targeted delivery and controlled release in response to changes in biological stimuli, such as pH, temperature, or redox potential, which trigger the release of the cargo (Zhang et al., 2023). Moreover, stimulus-responsive nanoplatforms, upon reaching the tumor site, can respond to endogenous stimuli and alter their structure or conformation, thereby initiating the release of loaded anticancer agents (Lu and Willner, 2015; Peng et al., 2022). By optimizing the controlled release system, the release rate of TPL can be regulated by nanocellulose to achieve sustained and stable drug release. A well-designed controlled release system allows nano-TCMs to adjust the drug release amount in response to changes in the internal environment, ensuring that the drug remains within an effective concentration range for an extended period. This approach not only enhances the therapeutic efficacy of the drug but also reduces the frequency of dosing and the medication burden on the patient. The reduction of toxic side effects has been effectively achieved through the introduction of nanotechnology. The emergence of targeted drug delivery systems (TDDSs) allows for the precise delivery of drugs to tumor sites, thereby minimizing drug accumulation in normal tissues and significantly reducing toxicity (Cai et al., 2021).

## Targeting strategies

The application of targeting strategies represents a significant advancement of nanotechnology in drug research and development. By coupling nanoparticles with specific antibodies or ligands, or by leveraging the unique microenvironment of tumor tissue, nanomedicines can accurately target lesions while minimizing damage to normal tissues. Current nanomedicine strategies are typically categorized into passive targeting and active targeting (Lammers, 2024). Active targeting involves custom surface modifications of nanoparticles with ligands that bind to specific receptors expressed on target cells (Spada and Gerber-Lemaire, 2025). This process is followed by receptor-mediated endocytosis, enabling nanoparticles to selectively accumulate in target cells. Once nanoparticles (NPs) are modified with various ligands, their internalization occurs through distinct pathways, thereby enhancing the uptake of nanomedicine by target cells. Clathrin-mediated endocytosis (CME) is a well-established transmembrane transport mechanism and plays a crucial role in receptor-mediated endocytosis (Sahay et al., 2010). The advantage of the active targeted drug delivery strategy lies not only in its ability to increase the concentration of the drug at the target site but also in its capacity to reduce the accumulation of the drug in non-target organs, thereby minimizing toxicity and enhancing efficacy (Li et al., 2021; Wei and Wang, 2025; Zhang X. et al., 2025). In contrast, passive targeting employs nanosized drug delivery systems (DDS) that utilize the enhanced EPR effect to facilitate drug accumulation in tumor tissue, thereby improving drug targeting (Belyaev et al., 2025; Sivasankaran et al., 2025). Passive targeting is predicated on the inherently small size of these systems and the EPR effect observed near tumor sites (Caro et al., 2021). Specifically, the rapid proliferation of tumor cells results in irregular neovascularization, leading to structural abnormalities in the vessel wall and increased microvascular permeability. Consequently, nanoparticles with reduced diameters can more readily traverse the vessel wall and penetrate tumor tissue (Fang et al., 2020). This phenomenon promotes the accumulation and prolonged retention of nanoparticles in the vicinity of the tumor site (Liu et al., 2024). Nanomedicine enables precise drug delivery through both active and passive targeting strategies. The active targeting approach involves modifying the surface of nanoparticles with specific ligands that bind selectively to receptors on tumor cell surfaces. In contrast, passive targeting of TCMs is facilitated by the enhanced EPR effect of nanocarriers, which allows for more efficient access to target areas (Li et al., 2018; Qiao et al., 2020). Additionally, certain nanocarriers can employ active localization strategies by binding to specific receptors, thereby enhancing the targeting capability of TCMs. Furthermore, nanocarriers can prolong drug release times, maintain controllable release profiles, and minimize toxicity, thereby maximizing therapeutic effects and improving the bioavailability of hydrophobic components of TCMs by enhancing their water solubility and stability (Lu et al., 2021; Wang et al., 2021; Wei et al., 2022). Additionally, the surface modification of nanoparticles decreases the interaction between drugs and the immune system *in vivo*, which in turn reduces the occurrence of immunogenic reactions. These advantages render nano-TCM safer and more effective for clinical applications. Guided by the principles of TCM, nanotechnology is poised to play a crucial role in the modernization and development of TCM.

## Challenges in clinical translation

The translation of nanomedicine from laboratory research to clinical application is constrained by multiple factors, primarily involving production processes, regulatory frameworks, material safety, and immunocompatibility. In terms of technical scalability, the precise fabrication of nanoparticles—such as control of size uniformity, impurity monitoring, and *in vitro–in vivo* correlation validation—places stringent demands on industrial-scale manufacturing (Csóka et al., 2021). Although emerging technologies such as microfluidics have improved production efficiency, challenges remain in terms of process stability and cost control (Adityan et al., 2020; Dash et al., 2020). On the regulatory front, existing evaluation frameworks are often inadequate to address the unique characteristics of nanomedicines. A key issue lies in the discrepancy between traditional “sameness”-based standards for generic drugs and the “similarity”-based assessment principles required for nanomaterials, leading to inconsistent approval outcomes among regulatory agencies such as the FDA and EMA. Moreover, the lack of reliable pharmacokinetic data and long-term toxicity profiles in clinical trials further complicates the approval process. Safety concerns are particularly evident in the unpredictable biological behaviors of nanocarriers, such as their ability to penetrate the blood-brain barrier and induce neurotoxicity (Szabat-Iriaka and Le Borgne, 2021), or cause oxidative stress and organ-specific damage due to surface charge or degradation byproducts (Scioli Montoto et al., 2020). Regarding immunocompatibility, surface modifications like PEGylation can prolong circulation time but may also trigger antibody-mediated accelerated blood clearance (ABC). Additionally, the formation of a protein corona may interfere with targeting efficacy and activate the complement system (Dash et al., 2020). Addressing these translational barriers requires the establishment of interdisciplinary collaboration mechanisms, including the development of standardized manufacturing techniques, globally harmonized regulatory guidelines, and fundamental research to elucidate the dynamic interactions between nanomaterials and biological systems.

## Mechanisms of nanoformulation TPL

### Anti-inflammatory

**FIGURE 2:**
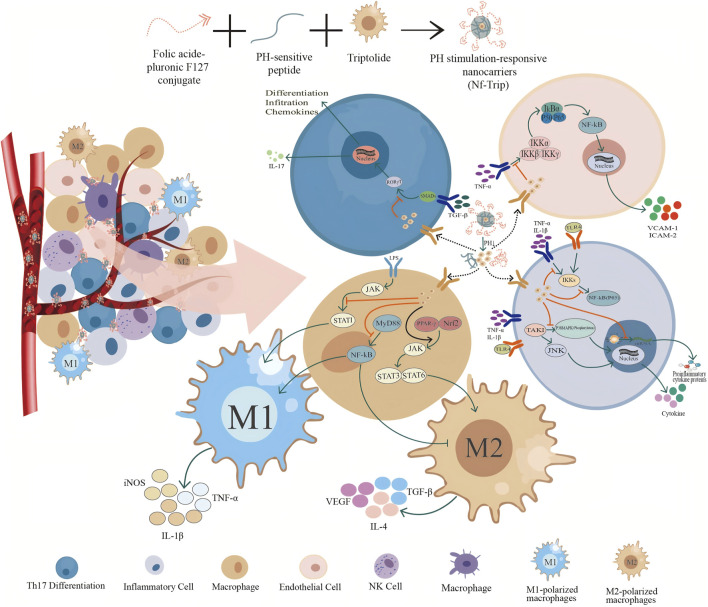
Intravascular release and mechanism of pH-responsive TwHF nanocarrier. Folate-conjugated F127, pH-sensitive peptides, and TwHF were co-assembled to construct a pH-responsive nanocarrier for the drug TwHF. In the *in vivo* environment, the TwHF nanoparticles are transported to inflammatory areas *via* blood vessels, where they disassemble due to the reduced pH at these sites. This process facilitates the release of TPL, allowing it to exert its anti-inflammatory and anti-tumor activities at the targeted locations. At inflammatory sites, TwHF exhibits various mechanisms of action on different cell types: it is absorbed by endothelial cells, inhibiting the release of adhesion factors VCAM-1 and ICAM-2 through the inhibition of the IKK signaling pathway, thereby reducing the adhesion of these factors to endothelial cells. In Th17 cells, it suppresses the transcription and translation of the pro-inflammatory factors IL-17 and the chemokine MMP-9 by inhibiting the SMADs and RORγT signaling pathways. In macrophages, TwHF promotes the differentiation of macrophages into M1 macrophages by inhibiting the JAK, STAT1, MyD88, and NF-kB signaling pathways. Concurrently, it enhances the activation of the JAK, STAT3, and STAT6 signaling pathways through the promotion of PPAR-γ signaling, further facilitating the differentiation of macrophages into M2 macrophages. In inflammatory cells, TwHF can inhibit the synthesis of pro-inflammatory cytokines and proteins by targeting IKKs, NF-kB (P65), and TAK1 signaling pathways, and by interfering with the translation program of RNA polymerase II.

Inflammation is a protective response that is generally regarded as a host defense mechanism against pathogen infections and tissue damage (Lian et al., 2021). However, when dysregulated, it can lead to immunopathology and further tissue damage (Gao et al., 2022). Inflammation is recognized as a significant contributor to the development of various diseases, including cancer, cardiovascular disease, diabetes, obesity, osteoporosis, RA, inflammatory bowel disease, asthma, and central nervous system-related disorders such as depression and Parkinson’s disease. Increasing evidence has elucidated how the dysregulation of inflammatory pathways can lead to numerous symptoms associated with chronic diseases (Laveti et al., 2013; Scarpa et al., 2024). Furthermore, inflammation plays a critical role in the pathogenesis of complex diseases and disorders, encompassing autoimmune diseases, metabolic syndrome, neurodegenerative diseases, cancers, and cardiovascular diseases (Chen and Yang, 2024; Cheng et al., 2025). Multiple cytokines and growth factors are present at sites of inflammation, each of which may influence the nature of the inflammatory response (Cifuentes et al., 2025). Cytokines serve as key modulators of inflammation, engaging in both acute and chronic inflammatory processes through a complex and sometimes contradictory network of interactions (Turner et al., 2014). The primary strategy for treating inflammation involves significantly alleviating disease severity by effectively inhibiting inflammatory processes, ultimately aiming to address the underlying disease. Research has demonstrated that TwHF can inhibit inflammatory responses and reduce cytokine levels, making it a focal point in the study of anti-inflammatory drugs. TwHF exhibits anti-inflammatory effects through multiple mechanisms. These include the inhibition of key pro-inflammatory factor expression, regulation of immune cell balance, suppression of antigen-presenting cell function, modulation of adhesion molecule and chemokine expression, and regulation of various signaling pathways (Figure 2).

Research on the anti-inflammatory applications of TPL utilizing nanotechnology demonstrates its inhibitory effects on the release of plasma inflammatory cytokines, including TNF-α(Mao et al., 2023), IL-1β, IL-6 (Chen et al., 2021), MCP-1 (Huang et al., 2021), MMP-3, MMP-9 (Piao et al., 2021), Cox-2, and NLRP3 (Fang et al., 2021), among others. This effect primarily stems from the inherent anti-inflammatory properties of TPL, which can be delineated in three key aspects: First, it interferes with the gene transcription of pro-inflammatory factors by inhibiting the activity of RNA polymerase I and II, thereby reducing mRNA production (Leuenroth and Crews, 2008; Wang et al., 2011). Second, it modulates the NF-κB signaling pathway, which is closely associated with inflammation, by inhibiting RNA polymerase II activity, leading to decreased NF-κB-mediated gene transcription and a reduction in the expression of pro-inflammatory factors such as VCAM-1, TGF-β, C3, and CD40. Finally, TPL may also influence the stability and translation of pro-inflammatory factor mRNA, further diminishing its protein synthesis and activity (Sun et al., 2011). The introduction of nanoformulations has significantly augmented the inhibitory effects of TPL. Specifically, nanoparticles, due to their high specific surface area and drug-loading capacity, can effectively enhance the concentration of the drug in target tissues or cells, thus achieving more efficient target binding. Furthermore, nanoparticles can regulate the drug release rate, prolonging its residence and action time within the body, thereby facilitating a sustained release effect. Additionally, the functional modification of the nanoparticle surface enables specific recognition and binding to inflammatory sites or cells, thereby increasing drug concentration in the inflammatory area and minimizing impacts and side effects on non-target tissues. Finally, by encapsulating drugs within nanoparticles, nanotechnology effectively protects them from degradation by the internal environment and enhances their stability.

TPL regulates the balance of immune cells through two primary mechanisms. First, it inhibits the production of pro-inflammatory T cells, such as Th17 cells, by suppressing pro-inflammatory cytokines and the transcription factor RORγT, which mediates the transcription of IL-17 (Jang et al., 2022). Second, it promotes the formation of anti-inflammatory T cells, such as Treg cells, by enhancing immunosuppressive functions and upregulating the transcription factor Foxp3. This balanced regulation of immune cells contributes to a reduction in the production of pro-inflammatory factors, thereby suppressing the inflammatory response. Additionally, nanoformulation enhances this regulatory mechanism by improving the targeting of TPL, controlling drug release, and protecting the stability of the drug, thereby allowing it to act more precisely on the relevant immune cells. Macrophages and dendritic cells, both classified as antigen-presenting cells, play a critical role in modulating the functions of other immune cells through the secretion of cytokines (Arora et al., 2020). Antigen-presenting cells, particularly dendritic cells, are essential for initiating immune responses by activating effector cells such as T cells *via* cytokine expression. Nano-formulated TPL has been shown to inhibit the function of these antigen-presenting cells and decrease cytokine expression, ultimately diminishing the intensity of the immune response. This inhibitory effect may occur through the disruption of differentiation, maturation, and migration processes of antigen-presenting cells. The use of nanoformulation significantly enhances the inhibitory effects of TPL by employing various mechanisms, including improved drug targeting, reduced distribution to non-inflammatory sites, and controlled drug release. This approach offers a novel foundation for developing anti-inflammatory treatments and introduces new strategies and methodologies.

The modulation of adhesion molecules and chemokines can be categorized into two primary aspects. First, the inhibition of adhesion molecules: these molecules are crucial in the inflammatory response, mediating the adhesion process between leukocytes and endothelial cells, thereby promoting the infiltration of inflammatory cells. Adhesion molecules facilitate the binding of leukocytes to endothelial cells (Zhang et al., 2017). TPL has been shown to significantly inhibit the expression of various adhesion molecules on endothelial cells, including vascular cell adhesion molecule-1 (VCAM-1) and intercellular adhesion molecule-1 (ICAM-1) (Liu et al., 2014). The reduced expression of these molecules diminishes the capacity of leukocytes to adhere to endothelial cells, consequently attenuating the inflammatory response. The second aspect involves the inhibition of inflammatory chemokine production: chemokines are small protein molecules that attract white blood cells to migrate to sites of inflammation. TPL can inhibit the production of various inflammatory chemokines, specifically by obstructing dendritic cell (DC)-mediated chemoattraction of neutrophils and T cells through the suppression of DC production of CC and CXC chemokines, such as MIP-1α, MIP-1β, and MCP-1, in response to lipopolysaccharide (LPS) (Liu et al., 2006; Yuan et al., 2019). These chemokines are integral to the recruitment of leukocytes during the inflammatory response; thus, their reduction aids in decreasing the infiltration of inflammatory cells and the overall extent of the inflammatory response. Furthermore, TPL in nanotechnology leverages the size effect and surface properties of nanoparticles to achieve passive targeted accumulation at inflammatory sites *via* mechanisms such as enhanced EPR. By exploiting the EPR effect, nanodrug delivery systems (NDDS) can passively concentrate drugs for improved tumor targeting, thereby increasing drug concentrations at inflammatory sites (Yu et al., 2021; Sun et al., 2022), which further amplifies the inhibitory effects on adhesion molecules and chemokine expression. In the context of nanotechnology, TPL more effectively inhibits the expression of endothelial cell adhesion molecules and inflammatory chemokines by increasing the drug concentration at the site of inflammation, thereby reducing the inflammatory response.

TPL can inhibit the inflammatory response by regulating signaling pathways associated with inflammation, such as the TLR4/MyD88 and MAPK signaling pathways. These pathways play crucial roles in the production of inflammatory factors and the activation of immune cells. Specifically, TPL may significantly influence the TLR4-NF-κB signaling pathway by inhibiting TLR4 expression, reducing the phosphorylation and degradation of IκB, and consequently inhibiting the activation of NF-κB (p65). This action leads to a decrease in cytokine production and alleviates the progression of acute lung injury (ALI) (Wang et al., 2014). Furthermore, nanoformulation enables the active targeted delivery of drugs through functional modifications of nanoparticle surfaces, such as the coupling of specific antibodies, peptides, or small molecule compounds. This targeted approach allows TPL to more accurately reach the inflammatory site and interact with key molecules in signaling pathways like TLR4 and MAPK. As a result, the concentration and duration of TPL at the inflammatory site are enhanced, increasing its effectiveness in regulating these pathways and inhibiting the production of inflammatory factors as well as the activation of immune cells.

### Anti-tumor

TwHF can achieve anti-tumor effects in the following ways: inhibit tumor cell proliferations and induce tumor cell apoptosis, inhibit tumor angiogenesis, and regulation of the tumor microenvironment. TPL induces tumor cell apoptosis through multidimensional and multitarget molecular mechanisms, involving mitochondria-dependent apoptotic pathways, death receptor signaling, ERS, oxidative damage, and crosstalk between key signaling pathways (Feng et al., 2024). TPL disrupts mitochondrial membrane potential by modulating Bcl-2 family proteins (e.g., upregulating Bax/Bak and downregulating Bcl-2/Bcl-xL), triggering cytochrome C release and subsequent activation of the caspase-9/caspase-3 cascade (Li X. et al., 2025). Simultaneously, TPL activates extrinsic apoptotic pathways *via* Fas/FasL and TRAIL/DR4/DR5 death receptor signaling, initiating FADD/caspase-8-dependent apoptosis. Furthermore, TPL-induced ROS burst not only activates the ASK1-JNK/p38 pathway by inhibiting the thioredoxin (Trx) system but also directly targets peroxiredoxin 2 (PRDX2), suppressing its antioxidant function to exacerbate ROS accumulation and mitochondrial/endoplasmic reticulum damage. ERS promotes Mcl-1 downregulation through the PERK/ATF4/CHOP axis while activating the calpain/caspase-12 pathway, forming synergistic crosstalk with mitochondrial apoptosis (Chen et al., 2024). TPL also inhibits NF-κB nuclear translocation to reduce the expression of anti-apoptotic genes such as XIAP and Survivin, while blocking pro-survival signaling pathways like PI3K-AKT and Src-ERK, collectively amplifying apoptotic effects (Li X. et al., 2024). Additionally, TPL-induced DNA damage (e.g., topoisomerase II inhibition causing double-strand breaks) and G1 phase cell cycle arrest (*via* p21 upregulation and CDK suppression) exacerbate genomic instability and proliferation inhibition (Zhang H. et al., 2024). In the cell division cycle, the checkpoint from G0/G1 to the S phase serves as a primary critical restriction (Nurse, 2000) (148). The complexes formed by cyclin D1 with CDK4 or CDK6, as well as cyclin E with CDK2, facilitate the transition of cells from the G0/G1 phase to the S phase by catalyzing the phosphorylation of Rb. TPL arrests the cell cycle in the G0/G1 phase, thereby inhibiting the proliferation of malignant glioma cells through the downregulation of cyclin D1, CDK4, and CDK6. Cell cycle regulation is a significant target for antiproliferative strategies in cancer treatment. Notably, TPL reverses the silencing of pro-apoptotic genes through epigenetic reprogramming and weakens cellular stress defense mechanisms by suppressing HSP70 expression. These mechanisms, combined with immunogenic cell death and chemosensitization effects, constitute a complex network underlying TPL-induced tumor cell apoptosis, highlighting its multitarget, multipathway antitumor properties.

VEGF is a crucial factor in angiogenesis, as it induces the proliferation and migration of endothelial cells and promotes the formation of new blood vessels. VEGF plays a significant role in tumor growth and metastasis. TPL has emerged as a promising antiangiogenic agent. The potent antiangiogenic effects of TPL are mediated through the inhibition of angiogenic pathways involving Tie2 and vascular endothelial growth factor receptor (VEGFR)-2 (Meng et al., 2014). TPL effectively inhibits angiogenesis both *in vitro* and *in vivo*, as evidenced by reduced endothelial cell proliferation, migration, network formation, and tumor angiogenesis. This inhibition occurs, in part, through the potent suppression of Tie2 and VEGFR-2 expression at both transcriptional and posttranscriptional levels, with a particular emphasis on VEGFR-2 production (He et al., 2010). The tumor microenvironment is a complex ecosystem comprising tumor cells, various immune cells, fibroblasts, and endothelial cells (Jiang et al., 2021). Tumor-associated macrophages (TAMs), which originate from circulating monocytes (Pechkovsky et al., 2010; Madsen et al., 2017), are recognized as key components of the tumor microenvironment. The observed increase in TAMs within this environment is closely linked to poor prognoses in cancer patients (Sunakawa et al., 2015; Wolf et al., 2015; Li, 2016; Lee et al., 2018; Yang et al., 2018). These findings demonstrate that M2-polarized macrophages (TAMs) can promote tumor growth *in vivo*, while also revealing that TPL-treated M2-polarized macrophages inhibit this growth-promoting effect. Study indicates that TPL selectively inhibits the functions of M2-polarized macrophages and TAMs (Li et al., 2020).

The application of TwHF nanotechnology has been significantly enhanced by the development of nanoscale vehicle-based systems for drug delivery. These advancements in nanoscience facilitate the precise distribution of medications to targeted tissues or cells, offering numerous benefits in the realm of targeted delivery. Key advantages include an improved ability to specifically target tissues or cells, the mitigation of chemo-resistance through intracellular transport, and the achievement of prolonged and regulated drug release (Brannon-Peppas and Blanchette, 2004; Mottaghitalab et al., 2015). Furthermore, nanoparticles can modify the pharmacokinetic and safety profiles of parent medications, allowing for targeted drug concentration at tumor sites and the regulation of drug release to maintain synergistic concentrations that enhance anti-cancer efficacy. Highly versatile nanoparticles have been engineered to respond to the tumor microenvironment or to be conjugated with ligands that specifically bind to cell-specific receptors, such as antigens, nucleolin, and folate receptors, representing the next-generation of nano-carrier drug delivery systems (Hrkach et al., 2012). The potential of TPL in anticancer therapy is not in doubt, and its multiple mechanisms make it an effective antitumor agent. Combined with the application of nanotechnology, it can not only improve its bioavailability, but also achieve targeted delivery and combination therapy, which provides a new idea for future cancer treatment.

## Potential application in prostate diseases

Prostate diseases, especially prostate cancer and benign prostatic hyperplasia, present unique challenges for treatment due to their dense stromal structure, complex microenvironment, and frequent development of drug resistance. The prostate, located deep within the pelvic cavity, is surrounded by physiological barriers such as the blood-prostate barrier, which limits the penetration and effective concentration of conventional drugs. Nanotechnology offers several distinct advantages in improving the efficacy and safety of therapeutic drugs for prostate diseases. Given the unique physiological structure of the prostate, nanotechnology can enhance drug delivery precision through both active targeting (e.g., using prostate-specific membrane antigen (PSMA) ligands) and passive targeting (e.g., EPR effect). Recent studies have leveraged the abnormal vascular structure of tumors, and the size advantage of nanoparticles (typically less than 200 nm), to achieve selective accumulation at prostate lesions *via* enhanced EPR. This passive targeting strategy not only increases the drug concentration at the lesion site but also significantly reduces systemic toxicity (Wang et al., 2023). Additionally, active targeting designs targeting prostate cancer-specific markers, such as PSMA, further optimize delivery efficiency. For instance, literature has reported that PDA-PEG-ACUPA nanomicelles achieve active targeting through the PSMA ligand ACUPA, significantly enhancing drug internalization in prostate cancer cells (Rosales-Barrios et al., 2025). This approach enhances therapeutic effects by increasing drug accumulation at the tumor site and reducing damage to normal tissues, thereby lowering systemic toxicity. On the other hand, the dense fibrous network in the prostate stroma has been a major barrier to drug penetration. Nanoparticles, with their unique physicochemical properties, can cross the stromal barriers through either transcellular or paracellular pathways, which can be facilitated by modifying surface charge or incorporating penetration peptides. This has led to the development of pH-responsive nanocarriers that can respond to specific signals in the tumor microenvironment. In this section, we will summarize the therapeutic mechanisms of TPL in prostate diseases and explore how nanotechnology can enhance the bioavailability of TPL and reduce its toxicity.

### Prostate cancer

Among males, prostate cancer (PCa) is the most prevalent solid organ malignancy and the second most common cancer worldwide (Dee et al., 2025). Particularly in China, both the morbidity and mortality associated with PCa have been increasing annually (Gandaglia et al., 2021). While the 5-year survival rate for patients with localized PCa is 99%, advanced PCa is typically incurable, with a 5-year survival rate of only 31%. Almost all prostate cancers that are incurable will progress from hormone-sensitive to castration-resistant prostate cancer within 2–3 years after medical treatments. At this stage, prostate cancer will become resistant to a range of therapeutic options until it advances to a stage where no effective drugs are available. In recent years, the significance of TCM in the management of prostate cancer, particularly as a first-line adjunctive therapy, has garnered increasing attention. Among these therapies, TwHF stands out as a distinguished representative. Studies have demonstrated that TPL possesses potent anti-tumor properties, effectively inhibiting the proliferation of prostate cancer cells and inducing their apoptosis. The anti-tumor effects of TPL can be attributed primarily to its capacity to induce cancer cell death, specifically through apoptosis. TPL has been shown to activate multiple apoptotic signaling pathways, including the endogenous apoptotic pathway. These pathways involve various apoptosis-related proteins, such as Bcl-2 family proteins and caspases, which promote the apoptosis of cancer cells (Huang et al., 2012; Zhao et al., 2016). Furthermore, TPL has been found to downregulate the expression of proteins associated with cell cycle regulation, including SUMO-specific protease 1, which is crucial for the growth and division of cancer cells. By inhibiting the expression of these key proteins, TPL effectively slows the proliferation rate of cancer cells. Additionally, some existing studies have indicated that TwHF can promote prostate cell apoptosis through multiple pathways. According to Huang et al., an active component extracted from Chinese medicinal herbs serves as an effective agent against prostate cancer. The anti-tumor activity of this component may be attributed to mechanisms involving the downregulation of SENP1, which restores the balance between SUMOylation and deSUMOylation, as well as the negative regulation of AR and c-Jun expression, thereby inhibiting AR and c-Jun mediated transcription in prostate cancer (Huang et al., 2012). Additionally, Han et al. report that TPL significantly inhibits the transactivation activity of both AR-FL and AR-Vs by disrupting the phosphorylation of AR at Ser515 through XPB/CDK7. Notably, TPL demonstrates effective anti-prostate cancer activities even at low doses. Furthermore, TPL synergizes with enzalutamide to reduce prostate cancer cell survival *in vitro* and enhances the anti-prostate cancer effect of enzalutamide on CRPC xenograft growth *in vivo*. These findings provide a strong rationale for further clinical evaluation of this combination (Han et al., 2017). The study by Zhao et al. suggests that TPL induces protective autophagy in prostate cancer cells *via* the CaMKKβ-AMPK signaling pathway (Zhao et al., 2016). Additionally, TPL has been shown to induce apoptosis by activating the functional p53 pathway in prostate cancer cells (Kiviharju et al., 2002). Yuan et al. also demonstrated that TPL strongly inhibits prostate cancer proliferation and suppresses cell migration and invasion, down-regulating the expression of Cav-1, CD147, and MMPs. Moreover, the combination of TPL and Cav-1 knockdown enhances the anti-migration and anti-invasion effects of TPL on prostate cancer cells (Yuan et al., 2016). Lastly, Chen et al. successfully designed a pH- and redox-responsive polymeric drug-coupled nanosystem for the co-delivery of DOX and TRI, facilitating the targeted rapid release of TwHF for prostate cancer treatment (Xu et al., 2018).

Given that cancers originate from alterations in biological processes at the molecular or nanoscale level, nanotechnology-based strategies represent an emerging and promising approach with substantial potential to enhance the diagnosis and treatment of cancer (Zhang Y. et al., 2025). The growth and deterioration of PCa are accompanied by a series of abnormal microenvironmental features, including hypoxia, low pH, increased ROS levels, elevated glutathione (GSH) concentrations, and overexpressed enzymes. Although these factors heighten the malignancy of cancer and complicate treatment, they also present potential avenues for diagnosis and the development of new treatment strategies for PCa(Hu et al., 2024). Nanoparticles can be engineered to create controlled release systems that respond to the characteristics of the tumor microenvironment, such as low pH, hypoxia, and high enzyme activity. In the acidic microenvironment of prostate cancer, nanoparticles can responsively release TPL, facilitating precise drug delivery and enhancing therapeutic efficacy. The occurrence and progression of cancer are marked by the overexpression of specific enzymes within the tumor, which can serve as triggers for enzyme-mediated drug release at the tumor site (Cordani et al., 2025). Upon reaching the tumor, these nanocarriers can respond to endogenous stimuli, altering their structure or conformation to initiate the release of loaded anticancer agents (Barve et al., 2014; Peng et al., 2022). Utilizing nanotechnology, TPL can exert its therapeutic effects on prostate cancer through targeted delivery, controlled release, and a combined mechanism of action. Prostate cancer cells may express specific receptors on their surfaces. By modifying the ligands of these receptors on the surface of nanoparticles, targeted delivery of TPL to prostate cancer cells can be achieved, thereby increasing the drug concentration at the tumor site while minimizing damage to normal tissues. Considerations such as antigen or receptor expression, the internalization of targeted conjugates, and the ability of nanomaterials to overcome drug resistance must be taken into account to create more efficient delivery systems. In this context, nanodevices, employing both passive and active targeting strategies, can significantly enhance the intracellular concentration of drugs in cancer cells, thus improving their efficacy while reducing toxicity to normal cells (Sanna et al., 2013).

TPL nanoparticles can also be utilized in conjunction with other therapeutic modalities, such as photodynamic therapy and chemotherapy. The synergistic effects of multiple treatment approaches facilitated by nanotechnology can further enhance the therapeutic efficacy against prostate cancer. Synergistic chemotherapy involves the simultaneous or sequential administration of multiple chemotherapy agents during treatment. In this context, it is essential to employ drugs with independent mechanisms of action. Combination chemotherapy utilizing a nanodrug delivery system can effectively overcome cellular resistance associated with monotherapy, while also minimizing drug dosage and associated side effects (Zheng et al., 2024). TPL can be utilized not only as a monotherapy but also in conjunction with other drugs to enhance therapeutic efficacy. Combination drug therapy is a clinically effective strategy employed to improve the efficacy of chemotherapy (Xu Z. et al., 2023; Zhang M. et al., 2024). Compared to single-agent chemotherapy, combination regimens can sensitize cancer cells to drugs, modulate various signaling pathways, and reduce the dosage of each drug, thereby minimizing side effects (Hu et al., 2016; Wu et al., 2023). Furthermore, combination therapy has been demonstrated to effectively overcome multidrug resistance (Qin et al., 2018; Hussain et al., 2025). For instance, the combination of TPL and 5-fluorouracil (5-FU) exhibits a more pronounced inhibitory effect on cancer cell growth than either agent alone (Liu, 2011). Given the androgen-dependent nature of PCa, chemotherapy agents are frequently combined with androgen inhibitors, such as docetaxel (DTX) and cabazitaxel (CBP) (Bouman-Wammes et al., 2018), or enzalutamide (Brasso et al., 2015). Chen et al. developed a prostate-specific membrane antigen (PSMA)-targeted combinatorial drug delivery strategy to co-deliver DTX and enzalutamide, thereby synergistically inducing apoptosis in PCa cells. This nanodrug delivery system demonstrated a higher uptake rate by cancer cells compared to single drug-loaded nanoparticles and free drugs, and exhibited superior inhibition of PCa cells relative to the control groups (Chen et al., 2022). Nanotechnology capitalizes on the unique prostate tumor microenvironment—characterized by hypoxia, acidity, and elevated protease activity—to enable precise drug delivery. Responsive nanocarriers, such as pH- or enzyme-activated systems, selectively release drugs like TPL at tumor sites while bypassing healthy tissues. Additionally, combinatorial strategiesovercome castration resistance by simultaneously targeting AR pathways and apoptosis, enhancing efficacy with minimized systemic toxicity. In alignment with the principles of drug combination therapy, a thoughtfully designed drug delivery system based on nanotechnology is anticipated to facilitate stable, controlled drug release and active targeting, thereby reducing cytotoxicity and enhancing the synergistic anticancer effects in the treatment of prostate cancer.

### Benign prostatic hyperplasia

Benign prostatic hyperplasia (BPH) is one of the most prevalent urinary diseases affecting men, particularly those over the age of 50, characterized by prostatic enlargement coincident with distinct alterations in tissue histology (Wang et al., 2017). The prevalence of this multifactorial condition rises with age. As men age, plasma testosterone levels decline, leading to a reduced testosterone-to-estrogen ratio. This change results in increased estrogen activity, which may promote the hyperplasia of prostate cells (Plochocki and King, 2022). Currently, TPL is utilized in the treatment of BPH by alleviating urinary tract symptoms and reducing prostate volume. Given the high prevalence of BPH among elderly men, TPL inhibits the progression of this condition through multiple signaling pathways. Prostatic hyperplasia often leads to issues such as frequent urination, urgency, and dysuria. TPL may help mitigate urinary tract symptoms associated with prostate hyperplasia by enhancing smooth muscle tone and reducing inflammation within prostate tissue. Additionally, preliminary studies indicate that TPL may contribute to a decrease in prostate volume by inhibiting the excessive proliferation of prostate cells. Although clinical evidence remains limited, this characteristic provides a theoretical basis for the use of TPL in managing BPH. The investigation of TPL in the context of prostate hyperplasia is still in its preliminary stages. Some existing animal studies suggest that TPL may have a beneficial effect on alleviating symptoms of BPH. For instance, a study examining the inhibitory effect of TPL on testosterone-induced BPH in male SD rats demonstrated that TPL could reduce prostate size in this model, as well as lower the levels of testosterone and DPH, and decrease serum PSA levels. Consequently, this suggests that TPL may inhibit the development of BPH(Wang et al., 2017). Furthermore, research on the anti-proliferative and pro-apoptotic activities of TPL (PG490) in primary cultures of human prostate epithelial cells revealed that TPL could induce cell senescence at low concentrations and trigger apoptosis through the regulation of signaling pathways at higher concentrations (Kiviharju et al., 2002). Therefore, future research should prioritize large-scale clinical trials to confirm the efficacy of TPL in the treatment of BPH.

### Prostatitis

Typically, in a normal physiological state, the body’s immune system is capable of precisely differentiating between its own tissues and external pathogens. However, when the immune system malfunctions or becomes aberrant, it might erroneously target the prostate tissue, misidentifying it as a foreign invader. This incorrect immune response subsequently leads to the occurrence of prostatitis. Prostatitis is a prevalent urinary tract condition that often leads to abnormalities in urination, including urinary urgency, frequent urination, odynuria, dysuria, and micturition difficulties. Additionally, it can result in pain in the suprapubic, lumbosacral, and perineal regions, as well as sexual dysfunction, collectively referred to as prostatitis syndrome. TPL exhibits anti-inflammatory and immunomodulatory effects on prostatitis. By inhibiting the release of specific inflammatory mediators, such as TNF-α and interleukins, TPL effectively reduces the inflammatory response in prostate tissue, thereby alleviating symptoms. TNF-α, primarily derived from mononuclear macrophages, is the earliest-secreted cytokine in the inflammatory response. It induces the production of chemokines, promotes the expression of adhesion molecules in epithelial cells and lymphocytes, and recruits inflammatory cells to the site of inflammation. Interleukin-17 (IL-17), a proinflammatory cytokine secreted by a subset of activated CD4 T cells, stimulates macrophages to secrete TNF-α and IL-1β, and encourages stromal cells to release inflammatory cytokines, chemokines, and growth factors (Zhang and Shen, 2025). IL-17 may contribute to the increased growth and metastasis of prostate tumor cells by either directly influencing prostate cells or indirectly elevating local levels of inflammatory cytokines and growth factors (Huang et al., 2025). The occurrence of prostatitis is closely linked to abnormalities in the immune system. TPL exerts an immunomodulatory effect, aiding in the restoration of immune balance within the body by regulating the function of immune cells, which may ultimately reduce both the incidence and recurrence rates of prostatitis. Immune cells play a crucial role in the body’s defense against external pathogens and the maintenance of internal homeostasis. The development of prostatitis may be associated with immune system disorders. Specifically, studies have shown that certain immune cells exhibit abnormal changes in activity and number throughout the pathological course of prostatitis. For instance, patients with chronic prostatitis demonstrate significantly increased numbers of specific immune cells, such as CD4^+^ T cells, which may exacerbate local inflammatory responses by releasing cytokines (Hou et al., 2009; Qu et al., 2024). Furthermore, TPL not only inhibits the secretion of DC chemokines but also suppresses T cell immune responses by reducing interactions among neutrophils, dendritic cells (DC), and CD4+/CD8+ T cells (Rao et al., 2022). There are limited prior studies on the treatment of prostatitis with TPL, primarily due to the toxic effects associated with this compound and its poor penetration into prostate tissue. However, with the advent of nanotechnology, nano-TwHF may offer significant potential in prostate treatment. Further experimental investigations are necessary to validate these findings.

## Future prospects

Nano-tripterygium effectively addresses the limitations associated with traditional TwHF, particularly in terms of toxicity—specifically hepatotoxicity and nephrotoxicity—as well as bioavailability. It offers significant advantages in enhancing drug targeting, achieving controlled release, and improving overall bioavailability. However, several challenges persist, including the necessity for stability studies, long-term safety evaluations, and large-scale production of nano-TPL. Future research on nano-TwHF should prioritize investigations into stability, drug release kinetics, immune response assessment, and clinical trials. In particular, further exploration is warranted regarding the physical and chemical stability of nano-TwHF under varying environmental conditions. Long-term safety studies of TwHF nanoparticles must evaluate potential long-term side effects and their implications for human health. Additionally, research should concentrate on elucidating the release kinetics of TPL nanoparticles *in vivo* to optimize both the rate and duration of drug release; this will ultimately enhance therapeutic efficacy. Evaluating potential immune responses induced by nano-TPL is crucial for developing strategies aimed at mitigating or preventing immune-mediated adverse effects. Finally, conducting clinical trials is essential to assess both the efficacy and safety of nano-TPL within real-world clinical settings. This will provide a scientific foundation for future applications.

## Conclusion

TPL has powerful anti-inflammatory and anti-tumor effects, but its clinical application is limited due to its own toxicity. With the progress of materials science, biomedical engineering and other disciplines in nanotechnology, nano-TPL has the advantages of significantly improving the stability and bioavailability of TPL drugs, inhibiting the proliferation of prostate cancer cells, inducing apoptosis, and regulating the tumor microenvironment and other ways to play a anticancer effect, while reducing the toxicity of drugs to liver, kidney and other organs. Nanotechnology in prostate diseases offers the unique advantages of enhanced targeting, improved drug solubility, and controlled drug release, which enhance therapeutic efficacy while minimizing side effects. Moreover, nanocarriers enable the co-delivery of multiple therapeutic agents, providing a multifunctional approach for treatment and diagnosis. It has a very good application prospect for a variety of diseases, including prostate diseases.

## Glossary

TPL: Triptolide
RA: rheumatoid arthritis
CRP: CRP
TwHF: Tripterygium wilfordii Hook F
NF-κB: Nuclear factor kappa B
NLRP3: NOD-like receptor family pyrin domain containing 3
STAT: Signal transducer and activator of transcription
TNF-α: tumor necrosis factor α
IL-1β: interleukin-1β
JAK: Janus kinase
MAPK: Mitogen-activated protein kinase
AKT: Protein kinase B
VEGF: vascular endothelial growth factor
MRSA: methicillin-resistant Staphylococcus aureus
P5CDH: Δ1-pyrroline-5-carboxylate dehydrogenase
ROS: reactive oxygen species
Tf-Tp@Lip: transferrin-modified liposomes
TCM: Traditional Chinese Medicine
EPR: enhanced permeability and retention
SLNs: Solid lipid nanoparticles
TDDSs: targeted drug delivery systems
MCP: Monocyte chemotactic protein
MMPs: Matrix metalloproteinases
CD: Crohn’s disease
DCs: Dendritic cells
NDDS: nanodrug delivery systems
ALI: acute lung injury
PARP: poly(ADP-ribose) polymerase
BPH: Benign prostatic hyperplasia
PSMA: prostate-specific membrane antigen
